# Prevalence of lichen planopilaris in the United States: A cross-sectional study of the All of Us research program

**DOI:** 10.1016/j.jdin.2022.05.003

**Published:** 2022-06-13

**Authors:** Tejas P. Joshi, Harrison Zhu, Zain Naqvi, Swathi Holla, Anthony Duruewuru, Vicky Ren

**Affiliations:** a School of Medicine, Baylor College of Medicine, Houston, Texas; b Department of Dermatology, Baylor College of Medicine, Houston, Texas

**Keywords:** epidemiology, hair loss, lichen planus, lichen planopilaris, prevalence

*To the Editor:* Lichen planopilaris (LPP) is a variant of lichen planus characterized by follicular hyperkeratosis and T-lymphocyte mediated perifollicular inflammation that often progresses to irreversible, scarring alopecia. There is a paucity of epidemiological data on LPP, with current estimates of LPP prevalence being based on single-center and single-city studies.^1^, ^2^, ^3^, ^4^ There are no studies that evaluate LPP prevalence in a diverse, nationwide cohort of American patients. Here, we aimed to estimate the prevalence of LPP using the All of Us database, a recently launched initiative by the National Institutes of Health that strives to include communities that have been historically underrepresented in research.^5^

We performed a cross-sectional analysis in All of Us and identified LPP cases using *International Classification of Diseases, Tenth Revision, Clinical Modification* code L66.1 and SNOMED code 64540004. Participants’ age, ethnicity, race, and sex were extracted from electronic medical records. The overall prevalence and prevalence within each age and racial/ethnic group were calculated using the Wald method with 95% CIs.

As of March 2022, All of Us has enrolled 327,654 participants. We identified 142 individuals with LPP, representing an overall prevalence of 0.043% (95% CI, 0.042-0.044). The average age at diagnosis was 62.4 years (SD, 11.2 years). The prevalence was highest in the 65-74 year age group (0.091%; 95% CI, 0.088-0.093), followed by the 55 to 64 year age group (0.054%; 95% CI, 0.052-0.055). Females constituted 91.6% of the LPP population. Prevalence in specific racial groups included 0.009% (95% CI, 0.007-0.011) in Asian, 0.029% (95% CI, 0.028-0.030) in Black, 0.021% (95% CI, 0.020-0.023) in Hispanic, and 0.057% (95% CI, 0.019-0.021) in White participants (Table I). Altogether, the majority of LPP cases were over the age of 55 years and self-identified as White (Fig 1).

**Table I:** Prevalence of lichen planopilaris in *All of Us* across age and racial/ethnic groups

Group	Total population, *n*	LPP cases, *n*	Prevalence, % (95% CI)	Female, *n* (%)
Overall	327,654	142	0.043 (0.042-0.044)	129 (91.6)
Age group, y				
<45	111,618	9	0.008 (0.008-0.009)	7 (77.8)
45-54	51,047	23	0.045 (0.043-0.047)	21 (91.3)
55-64	69,141	37	0.054 (0.052-0.055)	32 (86.5)
65-74	64,072	58	0.091 (0.088-0.093)	53 (91.4)
74+	36,480	15	0.041 (0.039-0.043)	15 (100.0)
Racial/ethnic group				
Asian	11,040	1	0.009 (0.007-0.011)	0 (0)
Black	69,087	20	0.029 (0.028-0.030)	18 (90.0)
Hispanic	60,540	12	0.020 (0.019-0.021)	12 (100.0)
White	177,647	101	0.057 (0.056-0.058)	93 (92.1)

*LPP*, Lichen planopilaris.

**Fig 1:**
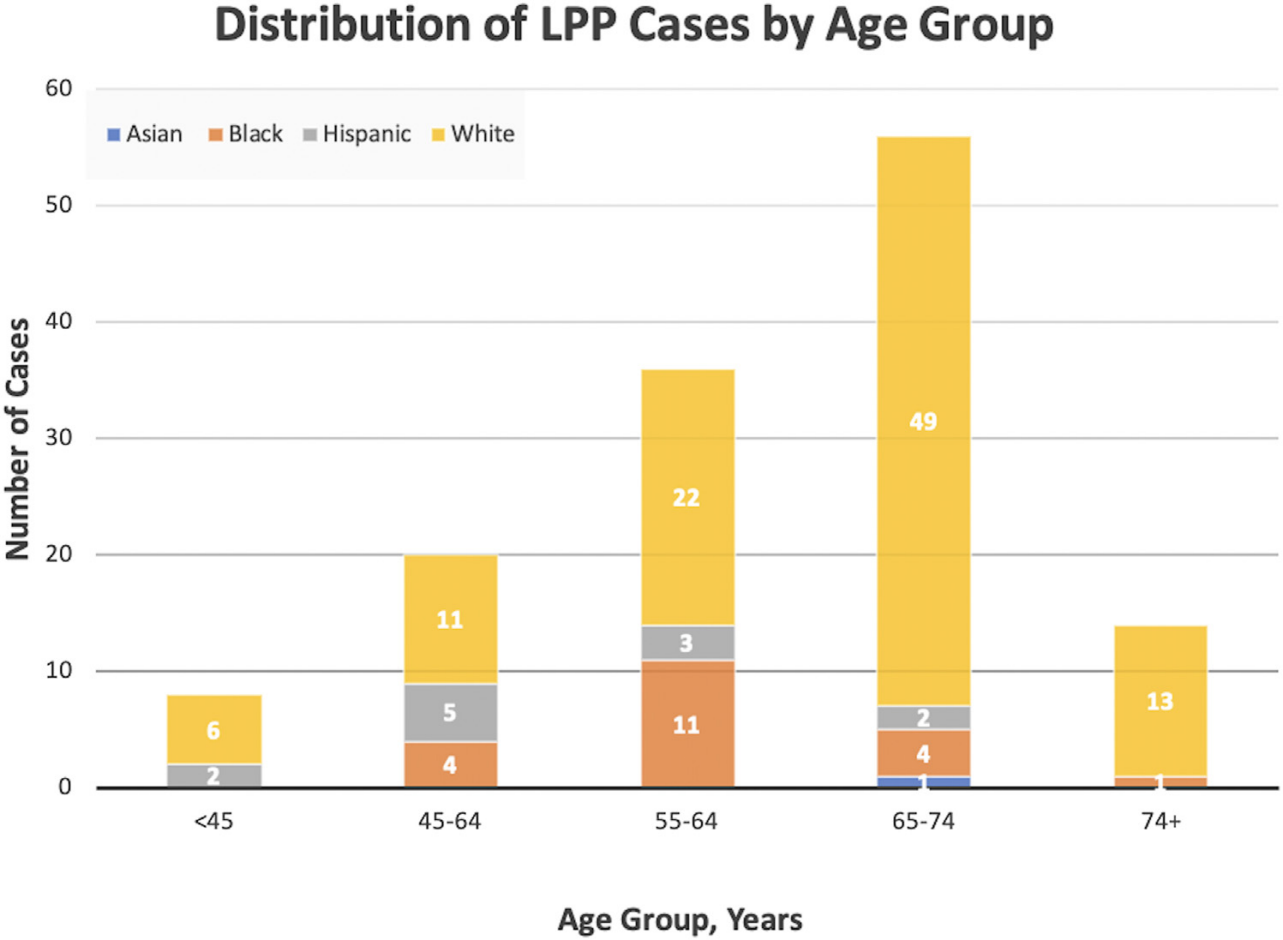
Distribution of lichen planopilaris cases by age group.

We show that LPP is most prevalent in women over the age of 55 years, a finding consistent with previous studies.^2^, ^3^, ^4^ In addition, we note LPP to be most prevalent among Whites, supporting the study by Trager et al.^4^ However, our prevalence estimate of 0.043% is higher than the 0.017% prevalence reported by Trager et al.^4^

Our analysis is subject to limitations. We base our identification of LPP cases on billing codes and are unable to confirm the diagnosis of LPP via histopathology reports. Additionally, older adults are overrepresented in All of Us, skewing the age distribution toward the 55 to 64 and 65 to 75 year age groups. Moreover, the diagnosis of hair disorders (in comparison to skin diseases) is often delayed, affecting the age at which diagnosis is thought to be most prevalent. Hair disorders may also be underdiagnosed in patients with skin of color; thus, the actual prevalence of LPP in patients with skin of color may be higher than the estimates we provide. Additional studies utilizing a national registry or a large insurance claims database would be helpful in further characterizing LPP prevalence in the United States.

## Conflicts of interest

None disclosed.
